# Research progress on the nonstructural protein 1 (NS1) of influenza a virus

**DOI:** 10.1080/21505594.2024.2359470

**Published:** 2024-06-25

**Authors:** Xiaoyan Zhang, Yuying Zhang, Fanhua Wei

**Affiliations:** a College of Animal Science and Technology, Ningxia University, Yinchuan, China; b School of Biological Science and Technology, University of Jinan, Jinan, China

**Keywords:** Influenza A virus, NS1, interferon, innate immunity, immune evasion

## Abstract

Influenza A virus (IAV) is the leading cause of highly contagious respiratory infections, which poses a serious threat to public health. The non-structural protein 1 (NS1) is encoded by segment 8 of IAV genome and is expressed in high levels in host cells upon IAV infection. It is the determinant of virulence and has multiple functions by targeting type Ι interferon (IFN-I) and type III interferon (IFN-III) production, disrupting cell apoptosis and autophagy in IAV-infected cells, and regulating the host fitness of influenza viruses. This review will summarize the current research on the NS1 including the structure and related biological functions of the NS1 as well as the interaction between the NS1 and host cells. It is hoped that this will provide some scientific basis for the prevention and control of the influenza virus.

## Introduction

Influenza viruses belong to the *Orthomyxoviridae* family and are negative-stranded RNA viruses with a capsid structure capable of infecting humans, birds, pigs, and dogs, and even whales, minks, and seals. The influenza virus genome is composed of eight segmented RNA fragments encapsidated by the nucleocapsid proteins (NP), each of which encodes at least one protein: polymerase A protein (PA), polymerase B1 protein (PB1), polymerase B2 protein (PB2), haemagglutinin (HA), neuraminidase (NA), NP, matrix proteins (M1 and M2), and nonstructural proteins (NS1 and NS2), *etc*. According to the different antigenicity of the M and NP, influenza viruses can be categorized into three types, namely type A, type B, and type C, as well as type D, which is a new type discovered in recent years. IAV can be further categorized into different subtypes according to the different surface glycoprotein HA and NA antigens, and 18 subtypes of the HA and 11 subtypes of the NA are known [1]. IAV is very harmful and widely spread, which not only brings huge economic losses to the livestock and poultry farming industry, but also can cross-species transmission of infection and death of mammals. The number of deaths due to influenza virus infection is between 290,000 and 650,000 per year in the whole world, which poses a great threat to the public health safety. There have been four influenza pandemics in history: the Spanish H1N1 pandemic in 1918, the H2N2 pandemic in 1957, the Hong Kong H3N2 pandemic in 1968, and the 2009 H1N1 pandemic [2]. Under immune pressure, the antigenic properties of influenza viruses are constantly mutating, thus escaping host immune surveillance and generating new pandemic strains. Currently, pandemic 2009 H1N1 and H3N2 are the main pandemic seasonal influenza virus subtypes. In recent years, the emergence of several avian influenza viruses and their worldwide pandemic have led to an increase in the number of human cases of avian influenza virus infection, exacerbating the public health risk of avian influenza viruses, especially the H5, H7 and H9 subtypes. Despite its low lethality in mammals and humans, H9N2 can act as an internal gene donor for H5N1, H7N9, and H10N8, leading to the possibility that it poses no less of a threat to humans than highly pathogenic avian influenza viruses [3,4].

## Structure of the IAV NS1

**Figure 1.**
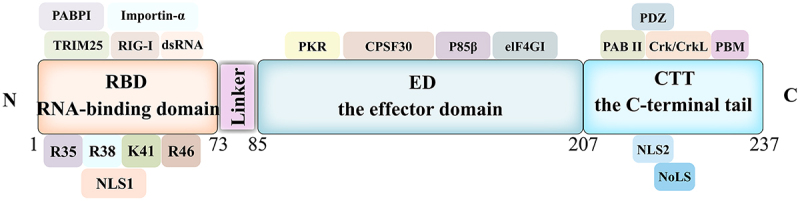
Structure of the influenza a virus NS1 protein.

The IAV NS1 is a determinant of virulence against host innate immunity and is highly conserved in structure [5]. It has an amino acid sequence length between 230 and 237, a molecular weight of approximately 28 kDa [6]. The NS1 is mainly localized in the nucleus, although considerable amounts of NS1 can also be found in the cytoplasm [5]. The NS1 protein consists of four distinct regions: the RNA-binding domain (RBD) located at the N-terminal end, the linker region, the effector domain (ED), and the C-terminal tail located at the C-terminal end [7–9]. The first 73 amino acids constitute the double-stranded RNA-binding domain, while the last 85–207 amino acids constitute the ED. The two domains are connected by a flexible linker region (LR). The last 30 residues form the C-terminal disordered tail. These are also described in Figure 1.

Schematic representation of the full-length NS1 structural domains and interacting proteins. NS1 consists of an RNA binding domain (RBD), a linker region (LR), an effector domain (ED), and a C-terminal tail (CTT).

The NS1 is a constitutive symmetric homodimer whose dimerization is mediated by high-affinity interactions between RBD, and its multimerization depends on the ED via W187 [10,11]. The RBD dimer consists of three α-helices from one monomer that interlocks with three α-helices from another monomer in a hexa-helix symmetric arrangement [12], where α2 and α2β form a “track” that interacts with the great groove of double-stranded RNA (dsRNA) [5,13]. The RBD structural domain binds dsRNA with low affinity and protects IAV from the antiviral response induced by IFN-α/β [5,13,14]. The various basic amino acids in the RBD (e.g. R35, R38, K41, R19, and R46) are essential for RNA binding activity and inhibition of the activation of the dsRNA-dependent protein kinase R (PKR) [15–18].

The LR domain, which consists of amino acid residues 74 to 85, can affect the relative spatial location of the RBD and the ED domains of the NS1, which is an important factor affecting pathogenicity [19]. Studies have shown that more than 90% of H5N1 influenza viruses lack 5 amino acids in the LR region, making the virus more virulent [20–23].

The structure of the ED domain monomer unit of the NS1 consists of three α-helices and seven β-chains. The protein core is arranged in a long central α-helix surrounded by a twisted antiparallel β-sheet [24]. The ED structural domain interacts with innate immune response host factors induced by influenza virus infection and plays an important role in RBD localization and nuclear export of NS1. ED also inhibits the antiviral response and promotes viral replication by targeting a variety of host factors, such as phosphatidylinositol-3 kinase (PI3K) and cleavage and polyadenylation specificity factor 30 (CPSF30) [25]. It has also been shown that the C-terminus of NS1 in avian influenza viruses (AIV) H5N8 clade 2.3.4.4 has an effect on the virus fitness of the host [26]. In addition, the C-terminal tail of the NS1 protein is an unstructured region, and the study of this domain is relatively weak, requiring further studies to explore its function.

The NS1 contains a nuclear localization signal (NLS) in the RBD. The NLS has an amino acid length between 35 and 41 residues, binds to importin-α, and facilitates translocation through the nuclear membrane [25]. The H3N2 and H2N2 viruses have a second NLS in the CTT, which also localizes the NS1 protein in the nucleus [27]. Co-existing with the C-terminal of NLS is a functional nucleolus localization signal (NoLS) [27], and the nuclear export signal (NES) is located within residues 138 to 147 [28]. The amino acids R35 and R46 within the RBD of the NS1 is highly conserved and interacts with the p85β regulatory isoform of PI3K to activate PI3K [15]. In addition, the NS1 enhances IRF3 activity by interacting with the p85β subunit, thereby increasing the production of IFN-I and pro-inflammatory cytokines [29]. The NS1 also inhibits the activation of the NF-κB pathway by targeting IKKβ and impairs the phosphorylation of histone 3 (H3) by IKK in the nucleus, thereby preventing the expression of antiviral genes [30]. The W187 residue of the ED domain mediates the formation of the NS1 protein dimers, and its mutation leads to a decrease in virulence [31]. In addition, residues E186, D189, and V194 play a crucial role in the binding of the NS1 to CPSF30, and amino acid variants at these sites results in restoring its ability to inhibit host gene expression [32].

## The functions of the IAV NS1

NS1 protein was first identified in 1971 and was widely known as an IFN antagonist. The function of NS1 protein is summarized in Table 1.

**Table 1.** A time line for the functional study of influenza virus NS1 protein.

Timeline	Functional research	Experimental cells	Strain type	References
1971	NS1 protein has been identified for the first time.	Hela-S3, BHK21-F, and MDBK cells.	WSN (Choppin, 1969)	[33]
1992–1999	NS1 protein binds to single-and double-stranded RNA.	293T cells	A/PR/8/34 (H1N1)	[14,34,35]
1994–2010	NS1 inhibits CPSF30-mediated pre-mRNA processing and nucleo-cytoplasmic transport of host mRNA.	HeLa, 293T, A549, MDCK, and PK-15 (swine) cells.	A/Udorn/307/1972, A/California/04/2009	[36–38]
1998	NS1 protein inhibits CPSF30-mediated pre-mRNA processing.	HeLa cells	A/Udorn/307/1972	[37]
2000–2009	The NS1 protein inhibits viral RNA-induced NF-κB activation by inhibiting RIG-I/TRIM25-mediated viral RNA sensing or by sequestering RNA from PKR.	MDCK, 293T, Vero, MEFs, A549, and L929 cells.	A/PR/8/34 (H1N1), A/Texas/36/1991 (H1N1), A/New Caledonia/20/1999 (H1N1), A/Wyoming/3/2003 (H3N2), A/Panama/2007/1999 (H3N2)	[16,39,40]
2006–2007	NS1 protein directly blocks the antiviral function of OAS and PKR.	MDCK and A549 cells.	A/Udorn/307/1972	[41,42]
2012	NS1 protein specifically inhibits IKK-mediated NF-κB activation.	A549, 293T, HeLa, and DF-1 cells.	A/WSN/33 (H1N1)	[30]
2016	NS1 protein interacts with NLRP3 to inhibit NLRP3 inflammasome activation.	293T, HeLa, MDCK, pDCs, and J774A.1 cells.	A/PR/8/34 (H1N1)	[43]
2019	NS1 targets the primary mRNA export receptor to block the nuclear export of the host mRNA.	A549 and HBEC cells.	A/WSN/33 (WSN), A/PR/8/34 (H1N1), A/Texas/36/1991	[44]
2020	NS1 inhibits RIG-I activation by degrading OTUB1.	Vero, MDCK, A549, 293T, and primary human lung epithelial cells.	A/PR/8/34 (H1N1), A/H1N1pdm09, A/Oklahoma/309/06 (H3N2)	[45]
2022	NS1 inhibits innate immunity by acting on β-transducin repeat-containing protein (β-TrCP)	A549, MDCK, and 293T cells.	A/California/04/09 (H1N1), A/PR/8/34 (H1N1)	[46]
2022	The interaction between the RBD domain of NS1 and TRBP affects the RNAi mechanism of the host.	293T cells.	A/PR/8/34 (H1N1), A/WSN/33 (H1N1)	[47]
2023	Mutations in the NS1 alter its function in regulating RIG-I-like receptor-mediated antiviral responses.	293T, A549, and MDCK cells.	A/PR/8/34 (H1N1), A/HangZhou/1/2013 (H7N9), A/Chicken/Jiande/09/2009 (H9N2), and A/pika/Qinghai/BI/2007 (H5N1)	[48]
2023–2024	NS1 cooperates with PA-X to mediate host shutdown and influence host innate immunity.	A549, and 293T cells.	A/PR/8/34 (H1N1)	[49,50]
2024	NS1 affects the expression of proteins involved in brain function.	Vero cells.	A/WSN/33 (WSN)	[51]
2024	PKR binds to N188, D125 and K126 of NS1, thus blocking the phosphorylation of PKR.	293T cells.	A/Udorn/307/1972	[52]
2024	NS1 mediates TGF- β activation to increase APEC adhesion to oviduct epithelium.	COEC cells.	A/chicken/Shaanxi/11/2012 (H9N2)	[53]

### The NS1 regulates the host fitness of IAV

Viruses can adapt to different host organisms because of their genetic variability and adaptability. The NS1 is one of the most polymorphic and mutagenic proteins in the IAV genome, and it plays an important role in the adaptation of influenza viruses to new host species. It has been found that the NS1 binds to CPSF30 to extensively inhibit host gene expression and their interaction is mainly through residues K108, D125, D189, F103, and M106 of the NS1 [38,54]. However, increasing evidence suggests that loss of CPSF30 binding may be selected to enhance influenza viral RNA replication and protein synthesis as the virus adapts to new hosts [55–57]. F103L and M106I mutations in the NS1 enhance influenza virus replication and virulence in BALB/c mice and promote early viral protein synthesis in MDCK cells in two different recombinant viral contexts. Moreover, these NS1 mutations enhance influenza virus replication in mouse cells pretreated with IFN-β [55]. Further studies show that there is an inverse relationship between the binding of functional CPSF30 and the activity of influenza viral polymerase [56]. However, the NS1 of A(H1N1)pdm09 strain undergoes 6 amino acid changes (E55K, L90I, I123V, E125D, K131E, and N205S) compared to the original protein, which restores the function of the NS1 protein in binding to CPSF30. The concomitantly mutated viral strain suppresses the host’s IFN and pro-inflammatory responses to a greater extent, but there is no significant increase in viral titre and the disease is partially attenuated [58].

Additionally, the NS1 plays an important role in influenza virus adaptation to new host species by inhibiting antiviral interferon signalling. It has been reported that the IAV NS1 contains a new domain of E96/E97 residues, which can mediate the interactions between the NS1 and the coiled-coil domain of TRIM25, thereby specifically inhibiting TRIM25 multimerization and RIG-I ubiquitination, and ultimately RIG-I signalling [40].

### The IAV NS1 regulates IFN signaling pathway

Innate immune response is the host’s first line of defence against viral infection which can limit viral replication in the early stages of infection through the interferon system [59]. The interferon system consists of three major classes, IFN-I, IFN-II, and IFN-III. IFN-I includes several isoforms, of which IFN-α and IFN-β are the most common. IFN-II is IFN-γ, and the number of IFN-III isoforms varies among host animals.

The innate immune system uses the pattern recognition receptor (PRR) between different cellular compartments to distinguish between microbial components that label the invading virus. The RIG-I like receptors (RLR) RIG-I and MDA5 can recognize pathogen-associated molecular patterns (PAMPS), including viral RNAs [60–66]. Activation of the RLR triggers the binding of mitochondrial antiviral signalling protein (MAVS), tumour necrosis-associated factor (TRAF), and TIR structural domain-containing aptamer for inducible IFN-β (TRIF), which in turn recruits the downstream signalling kinases TBK1, IKKε, and NF-κB. The transcription factors IRF3 and IRF7 are subsequently phosphorylated to form a dimer, which translocates to the nucleus to induce the production of IFN, mainly IFN-α/β [67]. IFN-α/β can bind to IFN-α/β receptors in infected cells or neighbouring cells and stimulate antiviral responses by autocrine and paracrine means [68]. Interaction of IFN-I with the receptor leads to phosphorylation and activation of the Janus family protein kinases (JAK) TYK2 and JAK1, which recruit and activate signalling molecules, including signal transducing and activating transcription factor 1 (STAT1) and STAT2 [68,69]. Upon activation, STAT1 and STAT2 form IFN-stimulated gene factor 3 (ISGF3) with IRF9, which translocates to the nucleus to induce the expression of interferon stimulated genes (ISGs) [69]. Products of ISG, such as myxoviral resistance (Mx), interferon-induced transmembrane proteins (IFITM), PKR, and 2”−5”-oligoadenylate synthase (OAS), induce host cells to enter into an antiviral state, which reduces the continued spread of virus from the initial site of infection [68,70–73]. Although the antiviral capacity of the host interferon system is very strong, influenza viruses can still escape the inner IFN-mediated immune response with the help of their viral proteins.

**Figure 2.**
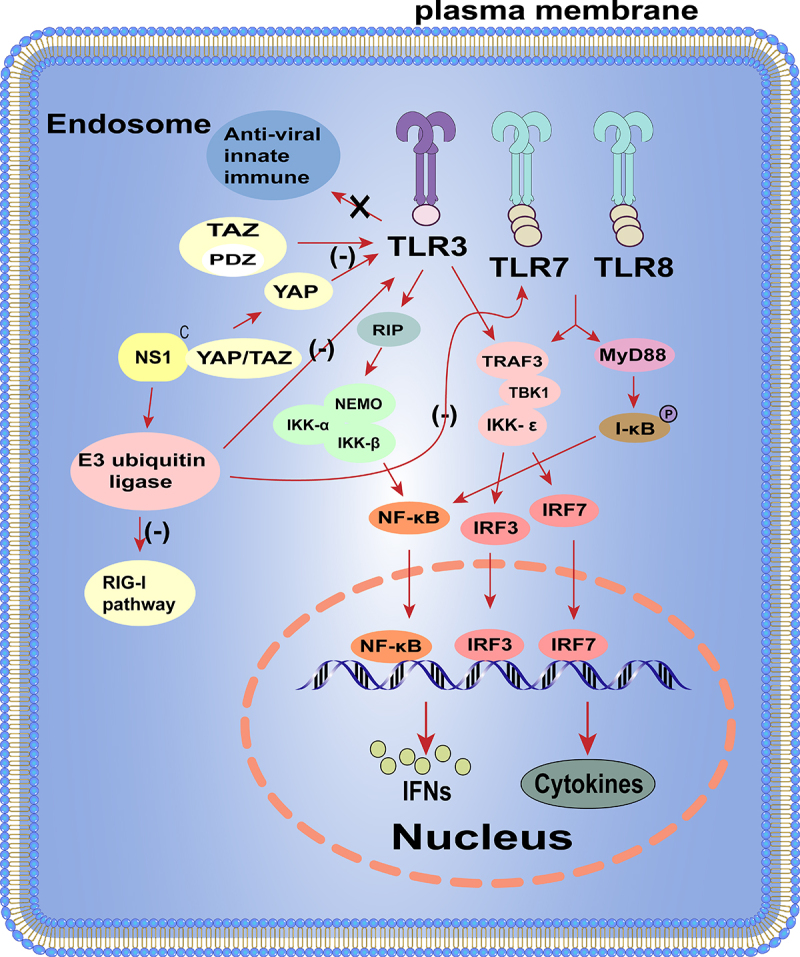
The NS1 protein regulates the IFN-I signalling pathway.

RIG-I is the main target for influenza virus infection *in vivo* and recognizes dsRNA or 5’triphosphate RNA (5′ppp-RNA) produced by virus replication in the cytoplasm [74–79]. The binding of viral RNA to RIG-I induces its conformational changes as well as ubiquitination and oligomerization of K63-linked RIG-I. Oligomerized RIG-I binds to MAVS, which in turn leads to oligomerization of MAVS and recruitment of TBK1 and IKKα/β. Subsequently, TBK1 phosphorylates the interferon regulatory factors (IRFs) leading to nuclear translocation of IRFs. At the same time, IKK-mediated phosphorylation and degradation of inhibitor kappa B (IκB) can lead to the release of NF-κB. IRFs and NF-κB form an active transcriptional complex that activates the expression of IFN-I [80]. Furthermore, influenza virus lacking the NS1 protein limits interferon production [81] and mutating NS1 protein inhibits interferon production and subsequently virus replication [82]. The IAV NS1 prevents viral and dsRNA-mediated activation of IRF3 and NF-κB, thereby enhancing viral replication and transmission [17,39]. In addition, binding of NS1 to MAVS promotes mitochondrial localization of NS1, which can inhibit MAVS-mediated IFN-I production [83]. These can be illustrated in Figure 2.

Like IFN-I, IFN-III is induced by viral infection. IFN-I and IFN-III are produced after recognition of viral ligands (e.g. nucleic acids) by multiple PRRs [84]. Studies have shown that the NS1 protein inhibits antiviral immune response by hijacking NF-κB-mediated transcription of IFN-III [85]. The NS1 directly inhibits IKK-mediated NF-κB activation and thereby attenuates antiviral gene expression [30], whereas inhibition of NF-κB leads to reduced expression of IFN-III [85].

#### The IAV NS1 regulates TLRs pathway

TLRs located on the cell surface or in the nucleus [86], and not only recognize viral infections and provides innate defence, but also play an important role in initiating and coordinating adaptive immunity [87,88]. Signaling from TLRs leads to upregulation of co-stimulatory molecules required for initial T cell activation and the production of pro-inflammatory cytokines [89]. TLR7 is originally identified to recognize imidazolines, which has antiviral and anti-tumour properties [90]. Influenza virus genomic single-stranded RNA (ssRNA) can be recognized by TLR7/8. After ssRNA is recognized, TLR7/8 recruits a TIR-domain acceptor, MyD88, which leads to phosphorylation and proteasomal degradation of IκB protein and release of NF-κB into the nucleus to regulate the expression of its target genes, including pro-inflammatory cytokines such as IL-6 and TNF-α, or IRF7 phosphorylation. Phosphorylated IRF7 forms a dimer and translocates into the nucleus to induce IFN-α and IFN-β gene expression [89]. Furthermore, TLR3 also acts as a PRR for sensing viral RNA and has emerged as a key intracellular receptor for influenza infection [91]. The main function of TLR3 is to induce the expression of IFN-I and pro-inflammatory cytokines in response to IAV infection [92]. Activation of TLR3 recruits TRIF through its own TIR structural domain, which in turn binds TRAF3/6 and then activates the TBK1/IKKε and IKK/IKK-β [93–96]. Ultimately, the transcription factors IRF3/7 and NF-κB translocate to the nucleus and cause activation, inducing type I IFN expression to prevent viral invasion [97].

**Figure 3.**
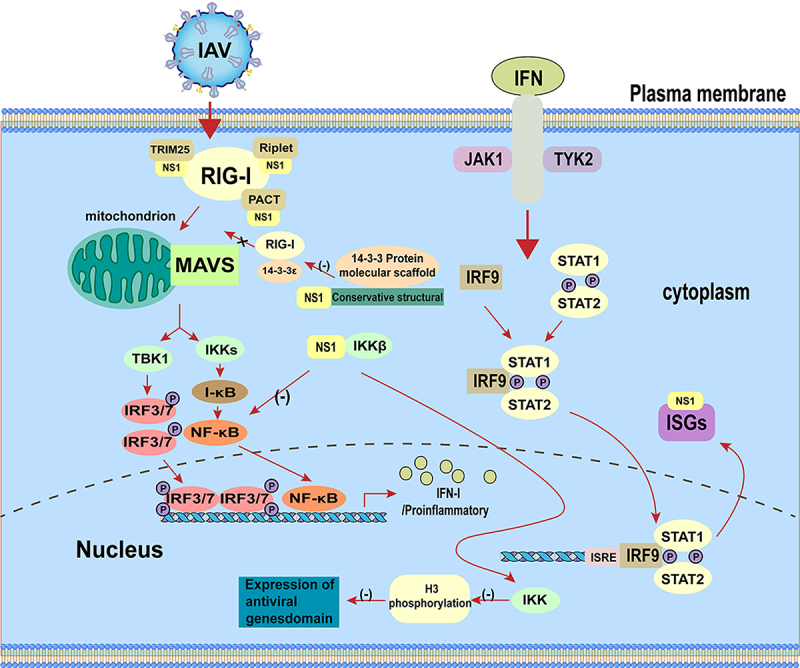
The NS1 protein regulates TLRs pathway.

It has been found that IAV can activate the Hippo effectors Yes-associated protein (YAP) and transcriptional coactivator with PDZ-binding motif (TAZ) through binding of the NS1 with C-terminal domain of YAP/TAZ, promoting their nuclear location. Moreover, YAP/TAZ downregulates the expression of pro-inflammatory and antiviral cytokines in response to IAV infection, facilitating viral replication and host cell apoptosis. In brief, the IAV NS1 hijacks cellular YAP/TAZ to promote its antagonistic effect on the host antiviral response by inhibiting TLR3 signalling [98]. In addition, studies have also revealed a novel NS1-mediated immune evasion mechanism in which the NS1 subverts the RIG-I, TLR3, and TLR7 pathways by targeting the E3 ubiquitin ligase TRAF3, which is a co-regulator linking these RNA-sensing pathways and IFN-I production [99]. These are also summarized in Figure 3.

The NS1 affects the TLRs pathway through various pathways thereby suppressing the innate immune antiviral response.

#### Influenza viral RNA recognition

The NS1 is a multifunctional virulence factor and can interact with different components of the IFN pathway to inhibit the host antiviral response. Upon influenza virus infection, RIG-I and TLRs can recognize and bind to specific sequences of influenza viral RNA, which can ultimately allow the IFN pathway to be activated and exert antiviral effects. At the same time, the NS1 protein can bind to influenza viral RNA through its RNA-binding domain located at the first 73 amino acids and thus prevents its recognition by TLRs and RIG-I. In addition, the NS1 protein competes with host factors (RIG-I, PKR, and OAS, *etc*.) to bind viral RNA and reduce its activity. For example, the NS1 inhibits OAS activation by binding to dsRNA and prevents RIG-I activation by binding and inhibiting TRIM25 activity [100]. Furthermore, IAV can utilize its NS1 protein to attenuate the antiviral response at the transcriptional level to ensure its replication [101]. In addition, the IAV NS1 also binds to a conserved structural domain in the molecular scaffold of the 14-3-3 protein, competing for the binding of RIG-I to 14-3-3ε and preventing RIG-I from translocating to the junctional MAVS towards the mitochondria, thereby inhibiting IFN-β expression [68,102].

#### The IAV NS1 disrupts the ubiquitin system

The ubiquitin-proteasome system, including ubiquitin, enzymes, and proteasomes, is key cellular processes involved in various pathways, including the innate immune response [68]. The UPS enhances and stabilizes the innate immune response and directly targets IAV proteasome-mediated degradation. In turn, influenza viruses hijack the UPS and use it to enter the cell and propagate, thereby disrupting the activity of the UPS and transforming it from an antiviral to proviral action [68]. Moreover, NS1 specifically targets UPS factors such as TRIM25, A20, OTUB1, MDM2, and the SUMOylation system [68]. The NS1 affects TRIM25 by interfering with its E3 ligase activity and by inhibiting IFN signalling activation [40]. The NS1 can also inhibit the RIG-I pathway by upregulating the expression of A20 (TNFAIP3), a negative regulator of the innate immune response [103,104]. OTUB1 is also a target of the NS1, although the mechanism has not yet been defined.

#### The IAV NS1 inhibits the processing of IFN-I precursor mRNA (pre-mRNA)

In addition to inhibiting innate immune pathways, the NS1 also suppresses host gene expression by blocking host mRNA translation (e.g. IFN-I and ISGs, *etc*.). The NS1 provides the critical function of blocking the processing of 3’ terminal cellular or viral precursor mRNAs by binding CPSF30 and polyadenylation-binding protein II (PABII). The NS1 binds to CPSF30 and prevents polyadenylation of host IFN pre-mRNA, leading to its accumulation in the nucleus and inhibition of IFN gene or IFN-related gene expression [74,105,106]. Polyadenylation of influenza viral mRNA is not affected, as it is dependent on viral polymerase [107]. The ability to bind CPSF30 also inhibits mRNA transport from the nucleus to the cytoplasm and mRNA splicing [108] and is different for different influenza viral strains. It has been demonstrated that the NS1 of H3N2 and H2N2 viruses are able to efficiently bind CPSF30 and inhibit IFN-I gene pre-mRNA processing, but the NS1 of H1N1/PR8 virus is unable to interact with CPSF30, and the L103F and I106N mutations increased the interaction of the NS1 with CPSF30, thus enhancing the pathogenicity of H5N1 viruses [109]. In addition, the NS1 protein can interact with the eukaryotic translation initiation factor eIF4B and participates in the translation initiation of host mRNAs. For example, the NS1-induced degradation of eIF4B leads to a decrease in the expression of IFN-induced transmembrane protein 3 (IFITM3), which in turn increases the level of viral replication [110]. The NS1 protein represses nuclear export of mRNA containing 3′ poly(A) and is directly involved in repressing host mRNA maturation by interacting with the poly(A) tail of mRNA [111]. Furthermore, the NS1 of H3N2 virus A/Udorn/72 can also inhibit pre-mRNA splicing *in vivo* and *in vitro* through association with the spliceosome and U6 small ribonucleic acid [111,112]. Through these multiple interactions with nuclear factors involved in mRNA maturation, the NS1 blocks the splicing and nucleoplasmic export of cellular mRNAs, thereby contributing to host shutdown. In conclusion, the extensive inhibition of host gene expression by the NS1 protein not only suppresses the efficient expression of IFN-I but also prevents the expression of other antiviral genes.

#### The IAV NS1 regulates the inflammatory response triggered by inflammatory vesicles

Influenza virus infection activates NLR family pyrin domain containing 3 (NLRP3) inflammatory vesicles and produces IL-1β production, thereby enhancing the host’s innate immune response. RNase L produces RNA fragments in the cytoplasm that also activate NLRP3 inflammasome [113] and RIG-I/MDA5 to induce IFN-α/β production [114]. NLRP3 is expressed in monocytes, macrophages, DCs, and neutrophils and is activated by cellular acidification induced by IAV ssRNA and newly synthesized M2 protein within the trans-Golgi network [115,116]. Activation of NLRP3 leads to the formation of a cytosolic multiprotein complex composed of oligomeric NLRP3, ASC and caspase-1 [117]. Caspase-1 then cleaves pro-IL-1β and pro-IL-18 to IL-1β and IL-18 pro-inflammatory cytokines, respectively [118].

It has been found that the NS1 and NLRP3 interaction inhibits the activation of inflammatory vesicles and reduces IL-1β production in mouse macrophage cell lines and human THP-1 cells [119]. The pH1N1/09 virus carrying C-terminal deletion of the NS1 induces higher levels of IL-1β than wild-type viruses, suggesting that the C-terminus of the NS1 protein has an inhibitory effect on the activation of inflammatory vesicles [119]. Further studies reveal that the RNA-binding structural domain (basic residues aa38 and aa41) and TRIM25-binding structural domain (acidic residues aa96 and aa97) of the NS1 protein are required for the inhibition of NLRP3 inflammatory vesicle activation. Notably, other influenza viral proteins also regulate NLRP3 inflammatory vesicle activation. For example, both M2 and PB1-F2 proteins stimulate oxidized DNA release and thus trigger NLRP3 activation, but the exact mechanism by which these viral proteins trigger NLRP3 activation remains unclear [117]. Therefore, a better understanding of the role of influenza viral proteins in regulating the inflammatory response and pathogenesis of IAV infection *in vivo* will helpful for developing more effective interventions to treat influenza virus-related diseases.

### The IAV NS1 regulates apoptosis in host cells

Apoptosis or programmed cell death, is a complex biological process. Apoptosis is activated mainly through the extrinsic apoptotic pathway (mitochondrial apoptotic pathway) and the intrinsic apoptotic pathway (death receptor apoptotic pathway). Apoptosis is a defence mechanism of host cells against IAV infection and can inhibit viral replication and propagation. However, influenza viruses have also evolved a variety of strategies to regulate host cell apoptosis to promote virus replication. In particular, the NS1 protein not only inhibits cell apoptosis in virus-infected cells, but also promotes host cell apoptosis after IAV infection [25].

Since viral replication must be completed before cells are disintegrated by apoptosis, expression of anti-apoptotic viral proteins can promote viral multiplication prior to host cell death [120]. Several studies show that influenza virus infection can induce apoptosis in a variety of host cells. The NS1 protein can inhibit the apoptotic process in IAV-infected host cells, and recombinant influenza viruses lacking the NS1 gene induce apoptosis in host cells than wild-type viruses [121]. The inhibition of host cell apoptosis by the NS1 protein may be related to the inhibition of IFN-I production, which can induce the onset of cell apoptosis.

**Figure 4.**
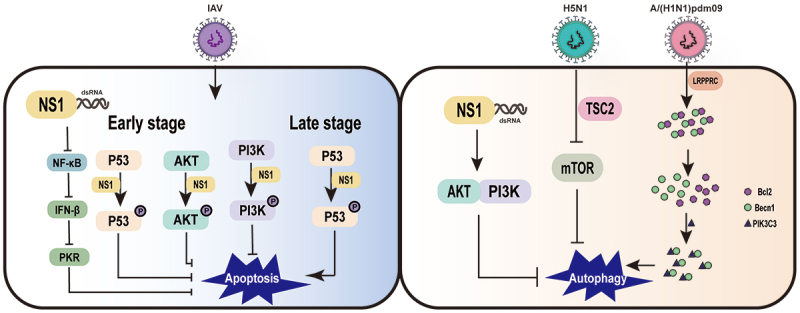
Influenza virus NS1 protein regulates cell apoptosis and autophagy in IAV-infected cells.

Influenza viruses inhibit host cell apoptosis mainly through upregulation of the anti-apoptotic PI3K/AKT pathway at the beginning of virus infection and by inhibition of the PI3K/AKT pathway and upregulation of the pro-apoptotic p53 pathway at the end of infection [121]. Studies have shown that viral proteins from different influenza virus strains regulate the apoptotic process of host cells differently, with some having pro-apoptotic effect and others playing anti-apoptotic functions. The balance between promoting and inhibiting host cell apoptosis is different during different stages of the influenza virus replication cycle [25]. It has been suggested that dsRNA can act as a pro-apoptotic factor in the early stage of IAV infection, but the presence of the NS1 prevents the activation of NF-κB, the synthesis of IFN-β [120], and the activation of PKR, thereby it shows an inhibitory state of host cell apoptosis [122]. Furthermore, the NS1 exhibits a complex relationship with host cell apoptosis, which depends on influenza virus subtype and cell type [25]. Infection with H5N1 subtype, a highly pathogenic influenza virus, is accompanied by a rapid development of primary pneumonia [123,124], which has also been shown to have an association with apoptosis [123,125]. The NS1 of H5N1 virus has an ability to rapidly induce apoptosis compared to all other subtypes [126]. This may be due to that H5N1 heterodimer of the NS1 can downregulate heat shock protein 90 (HSP90), which may play an important role in the induction of apoptosis [127]. The interaction between the NS1 and HSP90 induces a fragile interaction between Hsp90 and Apaf-1, but promotes the interaction between Apaf-1 and cytochrome C, which activates apoptosis associated with caspase-9 and 3 in A549 cells [128]. The NS1 can bind to the SH2 structural domain of the P85β regulatory isoform of PI3K or the N-terminal SH3 structural domain of Crk or CrkL during early and mid-infection [29,129–133], thereby activating the PI3K pathway to protect host cells from rapid apoptosis and provide sufficient time for viral replication [121,134–136]. However, the effect of the NS1 on the PI3K pathway depends on the viral strain, cell type, time course, and subcellular localization of the activation event [137]. For instance, the H3N2 NS1 antagonizes MAVS-mediated intrinsic apoptosis, the H9N2 NS1 suppresses Fas/FasL-mediated apoptosis [138], while the influenza/Swine/Colorado/1/1997 NS1 May induce apoptosis [139]. In addition, the ability of the NS1 protein to induce apoptosis in IAV-infected host cells is also dependent on the involvement of other influenza viral proteins or host factors. For example, p53 plays an important role in mediating apoptosis triggered by IAV infection, and the ability of the NS1 to bind to p53 and inhibit the p53-mediated apoptotic process in host cells suggests that p53 is one of the molecules to regulate apoptosis in IAV-infected host cells [140]. These are concluded in Figure 4. In summary, the NS1 protein plays an important role in apoptosis, thus targeting the NS1 protein at different stages of the viral life cycle provides a new idea for the treatment of IAV infection.

### The IAV NS1 affects cellular autophagy

Autophagy is a highly conserved intracellular lysosome-dependent process of self-digestion. Autophagy involves many kinds of signals, including endoplasmic reticulum stress, oxidative stress, and immune signalling activation. Autophagy is an effector of the immune system for the elimination of invading pathogens, and involved in the immune recognition of pathogen invasion. The autophagic process can be divided into two phases, the initial phase of autophagosome formation and the end effector phase (maturation), in which autophagic vesicles fuse with lysosomes and degrade autophagosome contents. Among them, PI3K-III, beclin (ATG6) and LC3 (ATG8) are important factors involved in autophagy [141].

In a variety of mammalian cell lines, IAV infection induces the accumulation of autophagosomes, suggesting that autophagy is involved in the IAV replication process. However, the role of autophagy in IAV replication is controversial and is related to cell type and viral strain [142]. In addition, autophagy is an important innate immune mechanism for host cells to inhibit IAV replication. IAV infection induces the formation of autophagosomes and, conversely, the degradation process of autophagosomes in influenza virus-infected cells, thereby limiting viral replication. This is due to the fact that blocking the degradation process of autophagosomes leads to the accumulation of autophagosomes and LC3-II in host cells and evades antigen recognition by viral proteins [143]. The negative stimulation of autophagy by NS1-deficient IAV may indicate that NS1 is involved in IAV-regulated autophagy. However, NS1 does not induce autophagy alone, and autophagosome formation requires the synthesis of HA and M2 [141]. The aggregation of LC3 in NS1 mutants (R38AK41A, Y89F) suggests that NS1 can utilize its dsRNA binding ability to activate PI3K-AKT and inhibit autophagosome formation [144]. In the early stages of IAV infection, NS1 assists M2-mediated autophagy by preventing phagocytosis of the viral RNP complex, thereby facilitating viral assembly [145].

It is currently believed that the M2, NP, HA and NS1 proteins of influenza virus are involved in the autophagic process. Both the M2 and NP proteins can induce autophagy through the AKT-mTOR pathway [142]. Cleavage products of the HA proteins of H5 and H7 subtypes can upregulate the level of LC3-II, indicating that the HA proteins can activate the autophagic process [141]. In addition, different subtypes of influenza virus have different effects on autophagy, for example, H5N1 virus can induce full and functional autophagy, while H9N2 and H1N1 viruses cannot induce functional autophagy. H5N1 virus inhibits cellular autophagy by suppressing the mTOR signalling pathway, a process that is dependent on the regulated expression of tumour suppressor protein 2 (TSC2) [146]. However, the A/(H1N1) pdm09 NS1 promotes viral replication by hijacking the IAV negative regulator leucine-rich pentatricopeptide repeat containing (LRPPRC) and their interaction leads to dissociation and degradation of Bcl2 from Becn1-Bcl2 heterodimer, which improves the interaction between Becn1 and PIK3C3, triggering autophagy [147]. These are also summarized in Figure 4. In conclusion, a better understanding of the process of autophagy regulation by IAV and the role of various viral proteins involved in autophagy regulation has important theoretical implications for the development of new antiviral drugs.

## Summary and outlook

Influenza viruses are highly contagious zoonotic pathogens that causes numerous deaths in humans and animals every year, posing a serious threat to public health safety. The emergence of avian and swine influenza virus has caused huge economic losses for farming industry. When influenza virus infects host respiratory cells, the inhibitory effect of the NS1 on the innate immune response of host cells determines the pathogenicity of the virus.

In order to elucidate the function of the NS1 protein in influenza virus infection and develop novel strategies for combating IAV infection, future research should focus on the detailed mechanism of the NS1 in inhibiting host innate immune response and the interaction of NS1 protein with host cellular components through various molecular biology techniques and proteomics approaches. Furthermore, the role of the NS1 protein in neuroleptic of influenza virus infection and in extracellular vesicles secreted by influenza virus-infected cells is also need to be further elucidated. In addition, studying the potential of the NS1 protein as targets for antiviral therapy and the development of live attenuated vaccines (LAV) will provide a certain theoretical basis for influenza prevention and control.

## Availability of data

Data sharing is not applicable to this article as no new data were created or analysed in this study.
